# Cotton stubble detection based on wavelet decomposition and texture features

**DOI:** 10.1186/s13007-021-00809-3

**Published:** 2021-11-02

**Authors:** Yukun Yang, Jing Nie, Za Kan, Shuo Yang, Hangxing Zhao, Jingbin Li

**Affiliations:** 1 College of Mechanical and Electrical Engineering, Shihezi University, Shihezi, 832000 Xinjiang China; 2 Industrial Technology Research Institute - XPCC, Xinjiang Production and Construction Corps (XPCC), Shihezi, 832000 Xinjiang China

**Keywords:** Machine vision, Visual defect detection, Stubble, Wavelet decomposition, Fusion feature, Texture feature

## Abstract

**Background:**

At present, the residual film pollution in cotton fields is crucial. The commonly used recycling method is the manual-driven recycling machine, which is heavy and time-consuming. The development of a visual navigation system for the recovery of residual film is conducive, in order to improve the work efficiency. The key technology in the visual navigation system is the cotton stubble detection. A successful cotton stubble detection can ensure the stability and reliability of the visual navigation system.

**Methods:**

Firstly, it extracts the three types of texture features of GLCM, GLRLM and LBP, from the three types of images of stubbles, residual films and broken leaves between rows. It then builds three classifiers: Random Forest, Back Propagation Neural Network and Support Vector Machine in order to classify the sample images. Finally, the possibility of improving the classification accuracy using the texture features extracted from the wavelet decomposition coefficients, is discussed.

**Results:**

The experiment proves that the GLCM texture feature of the original image has the best performance under the Back Propagation Neural Network classifier. As for the different wavelet bases, the vertical coefficient texture feature of coif3 wavelet decomposition, combined with the texture feature of the original image, is the feature having the best classification effect. Compared with the original image texture features, the classification accuracy is increased by 3.8%, the sensitivity is increased by 4.8%, and the specificity is increased by 1.2%.

**Conclusions:**

The algorithm can complete the task of stubble detection in different locations, different periods and abnormal driving conditions, which shows that the wavelet coefficient texture feature combined with the original image texture feature is a useful fusion feature for detecting stubble and can provide a reference for different crop stubble detection.

**Supplementary Information:**

The online version contains supplementary material available at 10.1186/s13007-021-00809-3.

## Background

The covering plastic film technique has the functions of protecting moisture, increasing temperature, inhibiting the growth of weeds and has been widely used since it was introduced into China.

As the main cotton-producing area in China, Xinjiang ranks first in the country in terms of the area and usage of covering plastic film, and the problem of residual film pollution is particularly severe.

At present, the residual film recovery procedure is mainly divided into the residual film recovery in late autumn, the residual film recovery before sowing, and the residual film recovery at the seedling stage. The residual film recovery in late autumn will not affect the harvest of crops and product quality. It is currently a more widely used method of recycling residual film, which is mainly divided into joint operation and segmented operation. The joint operation means that the machine can complete the task of stalk whipping and residual film recovery at one time after cotton harvest. The segmented operation is different. The cotton stalk whipping operation is first carried out. After the whipping is completed, the subsequent film recovery operation is performed. This type of machine has a simple structure, high reliability, and low energy consumption. However, the current segmented operation mainly relies on the manual operation of tractors to pull working tools. The working environment is dusty, and the work is monotonous. The driver’s continuous operation is labor-intensive, and it is easy to cause the missed of film recovery. The development of a navigation operation system for the residual film recovery can effectively reduce the labor intensity of the driver and significantly improve the production efficiency of the residual film recovery operation.

In agricultural machinery navigation operations, the global positioning system (GPS) has been used for many years [1–3]. In the case of low satellite signals or dynamic farmland environment, it is also important for the vision system to detect the navigation route in real-time to correct the deviation [4, 5]. In the agricultural field, the vision-based navigation route extraction algorithm gives priority to detecting the rows of crops, and extracting the route through the detected crop rows so that the working machine can automatically operate along the route. For the segmented residual film recovery operation, the stubble rows after the whipping maintain the characteristics of straight-line cotton planting. By detecting the stubble rows after whipping and adjusting the tractor to drive in real-time, it can ensure that the residual film recovery machine pulled by the tractor can collect the film along the film edge, and reduce the missed residual film.

The detection of cotton stubble rows is of great significance to developing the visual navigation system for segmented residual film recovery. In the existing studies of crop detection, the color, shape, and texture characteristics of the crop are usually extracted to segment it and recognize it.

In the study of using color for classification, Fu et al. [6] classified clustered kiwifruits by extracting the RGB and HSV color features of kiwifruit calyx. The accuracy rate reached 93.7%. Luo et al. [7] detected grapes based on HIS and YCbCr color space, they used an adaptive boosting (AdaBoost) classification algorithm, and the detection rate reached 96.56%. Fu et al. [8] proposed a detection based on S-V color features to detect bananas, and the detection rate reached 92.55%. For corn detection, Zheng et al. [9] extracted 14 vegetation indices related to color features to segment corn. The results showed that the 3-year total accuracy rate was 90.19%, 92.36% and 93.87%. The success rate of color-based detection largely depends on the studied crops and their color differences. When the colors of the crops and the background are very different, the color features may be helpful.

Shape features are another source for extracting information from images. Bakhshipour et al. [10] identified the crops in the sugar beet planting area based on the shape characteristics of the beet plants, evaluated the respective accuracy of the Support Vector Machine (SVM) and Artificial Neural Network (ANN) in the beet classification, and the accuracy rates of detecting sugar beets were 93.33% and 96.67%, respectively. Choi et al. [11] proposed a new navigation line extraction algorithm by using the morphological characteristics of rice leaves. Jahanbakhshi et al. [12] extracted the length, width, breadth, perimeter, elongation, compactness, roundness, area, eccentricity, centroid, nonhomogeneity, and width nonhomogeneity characteristics of carrots in the image to classify carrots. Kheiralipour et al. [13] introduced and extracted nonhomogeneity, and width nonhomogeneity characteristics to detect cucumber fruits. The shape feature can be used to detect crops. When the crops are not blocked or overlapped with each other, the detection accuracy can be very high.

Texture features play the vital role in image classification and image analysis. Zou et al. [14] segmented broccoli seedlings from weeds and soil by extracting gray-level co-occurrence matrix (GLCM) features and color features. When the training sample is greater than 50, the accuracy of the test set can reach 90%. Le et al. [15] used coefficient k-filter local binary patterns and contour masks (k-FLBPCM) to distinguish similar crops and weeds. Olaniyi et al. [16] used GLCM texture features to classify bananas automatically. When SVM was selected as the classifier, the accuracy reached 100%. Granito et al. [17] used GLCM and gray level run-length matrix (GLRLM) to classify weed seeds, the classification accuracy of the experimental data set is 99.2%. Guevara-Hernandez et al. [18] used GLCM and GLRLM algorithms to classify wheat and barley. The classification accuracy rates of bulk and single-grain samples are 98% and 68%, respectively.

The single feature extracted from the original image has limited abilities to classify images. Wavelet transform can make the features extracted from the image more diversified. Luo et al. [19] used a single-scale two-dimensional discrete wavelet transform to decompose and reconstruct the dried Hami Jujube image, extracting the reconstructed visual features for classification. To reveal the internal damage of the cylindrical shell more clearly, Parrany et al. [3] used a two-dimensional discrete wavelet transform as a post-processing technique to enhance the data. Thamizharasi et al. [20] used a weighting scheme to increase energy, they calculated the sub-band weights based on the energy and threshold of the wavelet decomposition sub-bands, multiplied the sub-bands, weights, and weighting factors to get a new sub-band, and created an enhanced energy discrete wavelet transform (DWT) image based on the new sub-band image. It is an effective method to extract features from the sub-bands of wavelet decomposition for image classification. Moreover, the decomposition effect of different wavelet bases is different. The selection of base wavelet is the main problem of using wavelet transform. Using different base wavelets on the same image may produce different results. Therefore, the selection of the wavelet base and the feature classification effect of decomposing sub-band extraction are the issues discussed later in this article.

As for the extracted crop features, the features input to the classifier can achieve the crops classification and detection. In the choice of the classifier, Supports Vector Machine (SVM) [5, 21], Artificial Neural Network (ANN) [22, 23], Random Forest (RF) [24, 25], and deep learning methods [26–28] are widely used. The segmentation algorithm based on deep learning can get a better segmentation effect, but it needs many training samples. Therefore, the application of deep learning algorithms in field stubble recognition is limited.

There is no relevant article report on cotton stubble detection research. For the cotton stubble detection tasks after harvesting and whipping, the color characteristics of the stubble are very similar to the disturbances such as broken cotton stalks, cotton shells, damaged leaves, and part of the field soil. These disturbances will obscure or extend the shape characteristics of the stubble. The selection of color features and shape features cannot segment the stubble well. In addition, the unevenness of the field during operation causes the camera to shake, while a single feature used for object detection can barely detect the cotton stubble in such a complex environment. Based on this background, this paper proposes a method for effectively detecting the cotton field stubble. The proposed method is based on a fusion feature of the original image texture feature, combined with the texture feature of the wavelet decomposition coefficient. The fusion feature can be effectively used for field stubble detection in the period of segmented residual film recovery in late autumn. This study provides a technical support for the residual film recovery navigation system. It also provides models and features suitable for stubble detection, which can be considered as a reference for other crops’ stubble detection and visual navigation operations. The article mainly completes the following three tasks:

1. Evaluate the stubble classification ability of the three texture features of GLCM, GLRLM, and LBP under SVM, ANN, and RF classifiers.
2. To extract the wavelet coefficient texture features after different wavelet base decomposition to classify the stubble, and to explore the classification effect of wavelet coefficient texture features after different wavelet decomposition.
3. To compare the classification effect of the original image GLCM texture feature combined with various wavelet coefficient texture features, and to verify the stubble detection effect of the fusion feature in different locations, different periods, abnormal driving and different algorithm.

## Methods

### Image acquisition

**Fig. 1:**
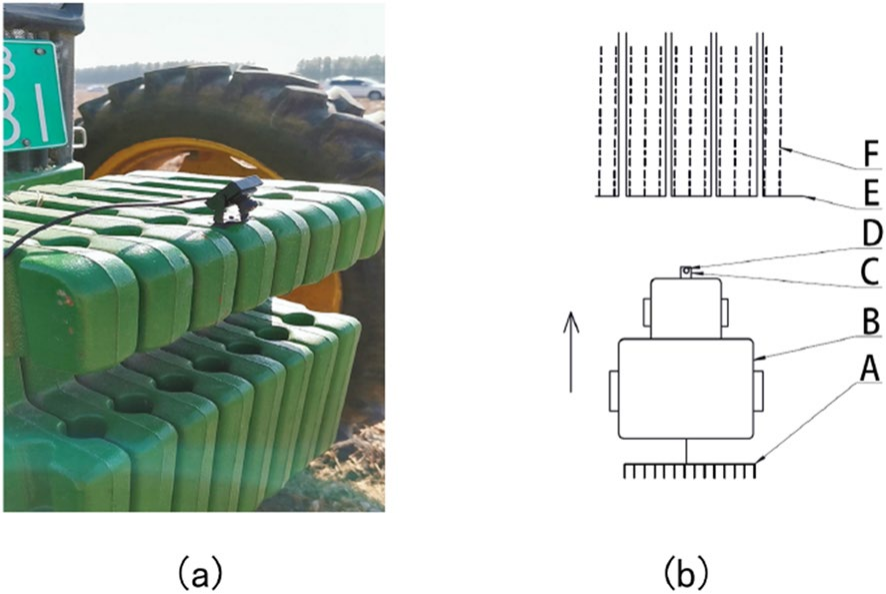
**a** The image of the camera installation; **b** the schematic diagram of the image acquisition process. (A) Residual film recovery equipment; (B) tractor; (C) counterweight; (D) camera; (E) residual film; (F) stubble rows

The camera for image acquisition is a Wild Forest wide-angle lens (130° wide-angle), and the output is the color image in.jpg format (RGB, 640 * 480 resolution). The computer processor used for image processing is Intel Core i7-7700K, the main frequency at 2.8 GHz, with Windows10, 64-bit operating system; The image processing software is PyCharm and OpenCV. In October 2019, images of the residual film recovery in the cotton field were taken in the 145th, 146th, and 152th regiments of the Eighth Division. The camera is installed on the front counterweight of the tractor, as shown in Fig. 1a. The schematic diagram of the image acquisition process is shown in Fig. 1b. The camera’s depression angles are 10°, 30°, and 45°, and the captured images include various situations such as sunny, cloudy, forward light, backlight, and abnormal driving.

### Manually segmented sample set

**Fig. 2:**
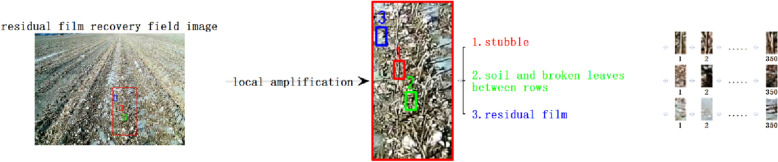
Schematic diagram of manually segmented sample set

The collected residual film recovery fieldwork image is shown in Fig. 2. The information such as stubble, soil, broken straw, broken leaves, and mulching film is included in the image. The above images can be divided into three types of representative images. The first type is stubble, the second type is soil and broken leaves between rows, and the third type is covering film. It is to manually segment the three types of sample images and calculate the relevant texture features as the input features of the classification model. The steps to segment the sample image are as follows: (1) set a 10 × 20 pixel marquee in PhotoShop software (the 10 × 20 pixel block can cover a complete stubble outline. If the selected pixel block is too small, only part of the stubble can be intercepted. Such images are similar to soil, broken leaves, and other features, which is not easy to classify. If the selected pixel block is too large, it will introduce too much noise and reduce the accuracy of classification). (2) Manually sample three types of targets from 350 sample images through this selection box in a random way, each type of image collects 350 samples. A total of 1050 pixel blocks are collected as sample pixel blocks, and the collection diagram is shown in Fig. 2. The sample-set is divided into a training set and test set according to the ratio of 8:2.

### Two-dimensional discrete wavelet

#### Transform (2D-DWT)

The two-dimensional discrete wavelet transform (2D-DWT) has a wide range of applications in the field of signal and image processing [29, 30]. Through Two-dimensional discrete wavelet transform (2D-DWT), the sample image can be decomposed into multiple sub-images with different resolutions, and high and low-frequency information is preserved. Better texture information can be extracted from high and low-frequency images [19]. If a continuous, square integrable function f(x) is given, its wavelet transform is composed of the inner product of f(x) and the real-valued wavelet function ψ_(x)_, the equation is as follows [31]:

1 $$W[f(s,\tau )] = < f,\psi_{s,\tau }^{k}> = \int_{ - \infty }^{\infty } f (x)\psi_{s,\tau }^{k} (x)dx$$

where $\psi_{s,\tau }^{k} \left( x \right) = \left( {\frac{1}{\sqrt s }} \right)\psi^{k} \left( {\left( {x - \tau } \right)/s} \right)$ belongs to the wavelet family, s ∈ Z, $\tau$, k ∈ {h, v, d} represent different resolution levels, and the direction parameters h, v and d represent the horizontal, vertical and diagonal directions, respectively. Now the two-dimensional wavelet decomposition is realized under s = 2^j^ and $\tau$ = 2^j^·n (j, n ∈ Z). It is to use wavelet function ψ_(x)_ and scale function φ_(x)_ to construct wavelet family and scale family:

2 $$\psi_{j,n}^{k} (x) = \frac{1}{{\sqrt {2^{j} } }}\psi^{k} \left( {\frac{{x - 2^{j} \cdot n}}{{2^{j} }}} \right),$$

3 $$\varphi_{j,n}^{k} (x) = \frac{1}{{\sqrt {2^{j} } }}\varphi \left( {\frac{{x - 2^{j} \cdot n}}{{2^{j} }}} \right).$$

The orthogonal basis of the subspace are related to the 2^j^ resolution. The wavelet atom is defined by scaling and translating with the three parent atoms of ψ^h^, ψ^v^, ψ^d^, which are calculated by ψ_(x)_ and φ_(x)_. The equations are as follows:

4 $$\varphi (x) = \varphi \left( {x_{1} } \right)\varphi \left( {x_{2} } \right),$$

5 $$\psi^{h} (x) = \psi \left( {x_{1} } \right)\varphi \left( {x_{2} } \right),$$

6 $$\psi^{v} (x) = \varphi \left( {x_{1} } \right)\psi \left( {x_{2} } \right),$$

7 $$\psi^{d} (x) = \psi \left( {x_{1} } \right)\psi \left( {x_{2} } \right).$$

**Fig. 3:**
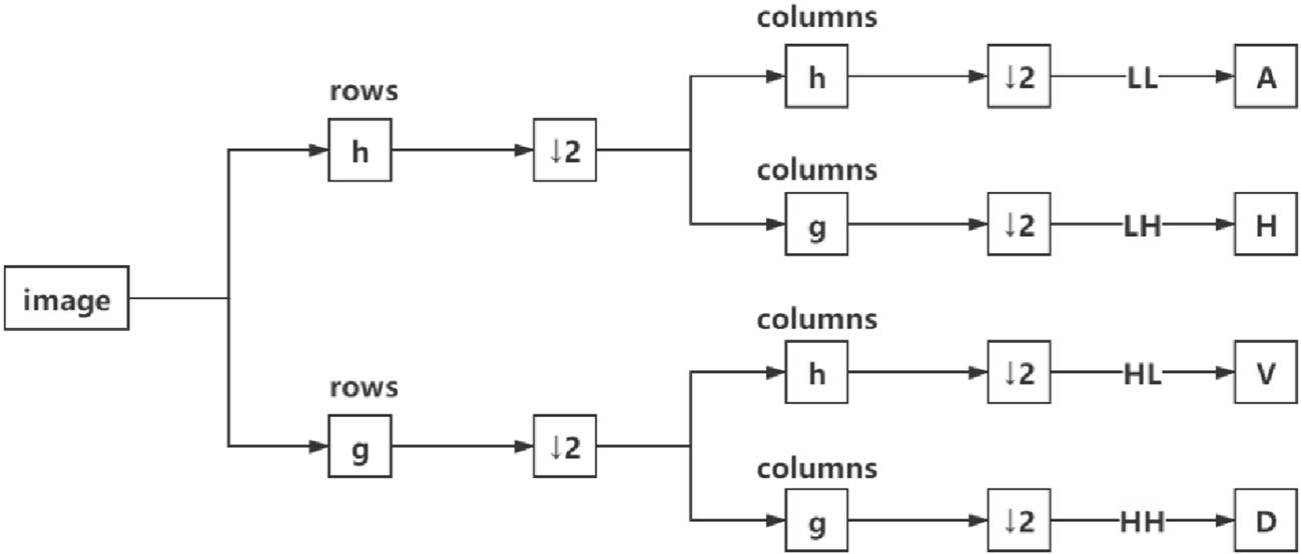
Schematic diagram of wavelet decomposition

The two-dimensional discrete wavelet transform (2D-DWT) is realized by the combination of digital filter and down-sampling. The digital filter is composed of high-pass (g) and low-pass (h) filters. Downsampling selects down two samples. As shown in Fig. 3, the original image is decomposed into four sub-band coefficients. H, V, D are the detail coefficients of the original image in the horizontal, vertical and diagonal directions, respectively. A is the approximate coefficient of the original image. The output results of image wavelet decomposition are four orthogonal sub-band components, such as low–low (LL), low–high (LH), high–low (HL) and high–high (HH), which correspond to sub-images A, H, V, and D, respectively.

**Fig. 4:**
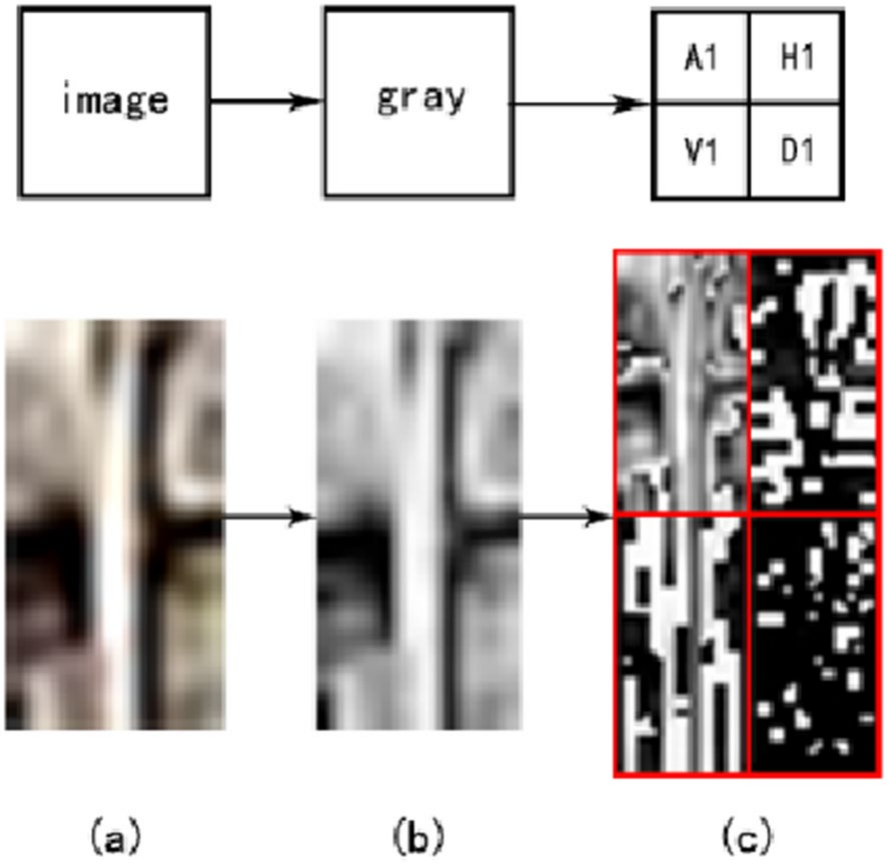
Four sub-bands of stubble after wavelet decomposition

Taking the stubble sample image as an example, Fig. 4a is the original stubble image, (b) is the gray image, (c) is the output four sub-band coefficients. The low-frequency component A1 reflects the contour of the image, and the high-frequency components H1, V1, D1 reflect the horizontal, vertical, and diagonal details of the image.

### Feature extraction

It is to extract the features of the three types of sample images to classify the images, mainly discuss the effect of texture features on the stubble detection, and select three texture features based on GLCM, GLRLM and LBP for subsequent research.

#### (1) Gray level co-occurrence matrix (GLCM)

**Fig. 5:**
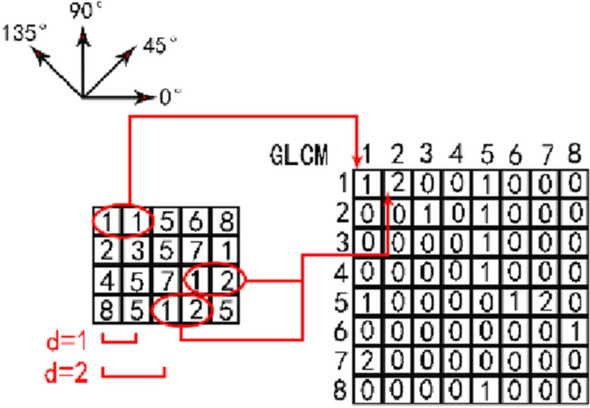
Principle of GLCM

Texture features can be described as the surface and structural properties of an image. In the visual system for detection and classification, texture plays a vital role. The value of GLCM is expressed by the frequency of pixel pairs appearing in distance d and direction θ [32]. Under normal circumstances, θ will take the values of 0°, 45°, 90° and 135°. If the image has k gray levels, the size of the co-occurrence matrix is k * k. The principle is shown in Fig. 5. The left side is the grayscale image of the original image. The right side is the corresponding GLCM. There are 8 Gy levels in the original image, so the size of the GLCM is 8 * 8. Taking the direction 0° and the distance d = 1 as an example, the 0° direction (1, 1) appears once in the original image, and the corresponding GLCM (1, 1) is 1. The 0° direction (1, 2) appears twice in the original image, and the corresponding GLCM (1, 2) is 2. By analogy, the corresponding GLCM can be obtained.

It is to select six types of GLCM statistics features as the classification features of the image, which are Angular Second Moment (asm), Entropy (ent), Correlation (cor), Dissimilarity (dis), Contrast (con) and Homogeneity (hom). The calculation equation is shown in Table 1. The G(i, j) is the value of the GLCM in the i-th row and j-th column, k is the number of gray levels in the GLCM, u and s are the mean and variance of the GLCM. For the three types of sample images, the size of the ROI we need to process is 10 * 20 pixels, the gray level is 256, the step size is 1, the angle is 0°, 45°, 90° and 135°. To calculate the corresponding GLCM, we can get a 256 * 256 GLCM, and each type of image will finally produce 24 (4 * 6) dimensional features as the GLCM texture feature of the sample image.

**Table 1:** The calculation equations for the characteristics of the GLCM

Feature	Equation
asm	$asm = \sum \limits_{i = 1}^{k} \sum \limits_{j = 1}^{k} (G(i,j))^{2}$
ent	$ent = - \sum \limits_{i = 1}^{k} \sum \limits_{j = 1}^{k} G(i,j)\log G(i,j)$
cor	$cor = \sum \limits_{i = 1}^{k} \sum \limits_{j = 1}^{k} \frac{{(ij)G(i,j) - u_{i} u_{j} }}{{s_{i} s_{j} }}$
dis	${\text{dis}} = \sum \limits_{i = 1}^{k} \sum \limits_{j = 1}^{k} G(i,j)|i - j|$
con	$con = \sum \limits_{{{\text{n}} = 0}}^{k - 1} n^{2} \left\{ { \sum \limits_{i = 1}^{k} \sum \limits_{j = 1}^{k} G(i,j)} \right\},|i - j| = n$
hom	$hom = \sum \limits_{i = 1}^{k} \sum \limits_{j = 1}^{k} \frac{G(i,j)}{{1 + (i - j)^{2} }}$

#### (2) Gray level run length matrix (GLRLM)

**Fig. 6:**
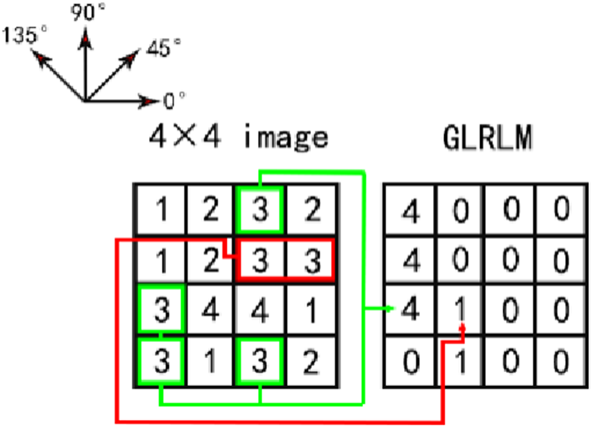
Principle of GLRLM

The gray level run length matrix (GLRLM) is the second texture extraction algorithm in this article [33]. The size of the GLRLM is (M × N), where M is equal to the maximum gray level, and N is the maximum run length possible in the corresponding image. Typical directions are 0°, 45°, 90° and 135°. To calculate the run length in each direction will generate the corresponding GLRLM. In GLRLM (i, j), i is the gray value, j is the number of consecutive occurrences of the gray value. In the statistics, the gray value of the maximum run length starts to be counted. For those gray values that have been counted, they will not be included in the statistics for the next run length. The schematic diagram of the principle is shown in Fig. 6, the left side is the original image. The right side is the GLRLM in the 0° direction. Taking gray scale 3 as an example, when the run length is 4 and 3, the gray scale 3 does not exist, that is, GLRLM (3, 4) and GLRLM (3, 3) = 0. When the run length is 2, the gray scale 3 has appeared once, as shown by the red box in the Fig. 6, that is, GLRLM (3, 2) = 1. When the run length is 1, the gray scale 3 has appeared 4 times, as the green box in the Fig. 6, GLRLM (3, 1) = 4.

Table 2 shows the eleven statistical characteristics and equations based on the GLRLM in this article. Eleven features of the short run, long run, gray level non-uniformity, run length non-uniformity, run ratio, low gray level run, high gray level run, short run low gray level, short run high gray level, long run low gray level, long run high gray level are extracted. The directions are 0°, 45°, 90° and 135°. Each image can generate 44 features (11 * 4), where Q(i, j) is the GLRLM, i is the gray value, j is the run length, and S is the sum of all values in the GLRLM.

**Table 2:** The calculation equations for the characteristics of the GLRLM

Feature	Equation
Short run	$sr = \sum\limits_{i} {\sum\limits_{j} {{{\left( {Q(i,j)/j^{2} } \right)} \mathord{\left/ {\vphantom {{\left( {Q(i,j)/j^{2} } \right)} S}} \right. \kern-\nulldelimiterspace} S}} }$
Long run	$lr = \sum\limits_{i} {\sum\limits_{j} {{{\left( {j^{2} Q(i,j)} \right)} \mathord{\left/ {\vphantom {{\left( {j^{2} Q(i,j)} \right)} S}} \right. \kern-\nulldelimiterspace} S}} }$
Gray level non-uniformity	$gln = {{\sum\limits_{i} {\left( {\sum\limits_{j} {Q(i,j)} } \right)}^{2} } \mathord{\left/ {\vphantom {{\sum\limits_{i} {\left( {\sum\limits_{j} {Q(i,j)} } \right)}^{2} } S}} \right. \kern-\nulldelimiterspace} S}$
Run length non-uniformity	$rln = {{\sum\limits_{j} {\left( {\sum\limits_{i} {Q(i,j)} } \right)}^{2} } \mathord{\left/ {\vphantom {{\sum\limits_{j} {\left( {\sum\limits_{i} {Q(i,j)} } \right)}^{2} } S}} \right. \kern-\nulldelimiterspace} S}$
Run ratio	$rr = \sum\limits_{i} {\sum\limits_{j} {{S \mathord{\left/ {\vphantom {S {jQ(i,j)}}} \right. \kern-\nulldelimiterspace} {jQ(i,j)}}} }$
Low gray level run	$\lg r = \sum\limits_{i} {\sum\limits_{j} {{{Q(i,j)} \mathord{\left/ {\vphantom {{Q(i,j)} {Si^{2} }}} \right. \kern-\nulldelimiterspace} {Si^{2} }}} }$
High gray level run	$hgr = \sum\limits_{i} {\sum\limits_{j} {i^{2} {{Q(i,j)} \mathord{\left/ {\vphantom {{Q(i,j)} S}} \right. \kern-\nulldelimiterspace} S}} }$
Short run low gray level	$srlg = \sum\limits_{i} {\sum\limits_{j} {{{Q(i,j)} \mathord{\left/ {\vphantom {{Q(i,j)} {Sj^{2} i^{2} }}} \right. \kern-\nulldelimiterspace} {Sj^{2} i^{2} }}} }$
Short run high gray level	$srhg = \sum\limits_{i} {\sum\limits_{j} {i^{2} {{Q(i,j)} \mathord{\left/ {\vphantom {{Q(i,j)} {Sj^{2} }}} \right. \kern-\nulldelimiterspace} {Sj^{2} }}} }$
Long run low gray level	$lrlg = \sum\limits_{i} {\sum\limits_{j} {j^{2} Q(i,j)Si^{2} } }$
Long run high gray level	$lrhg = \sum\limits_{i} {\sum\limits_{j} {i^{2} {{Q(i,j)} \mathord{\left/ {\vphantom {{Q(i,j)} S}} \right. \kern-\nulldelimiterspace} S}} }$

#### (3) Local binary pattern (LBP)

**Fig. 7:**
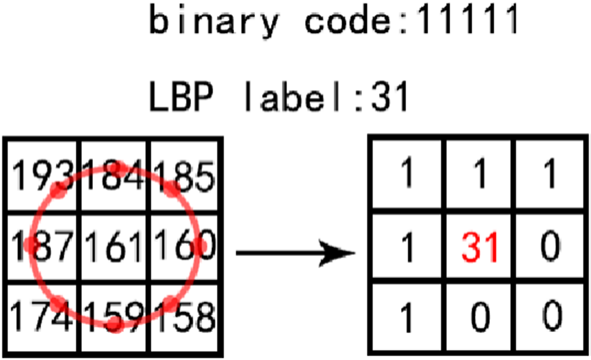
Principle of constant mode LBP

**Fig. 8:**
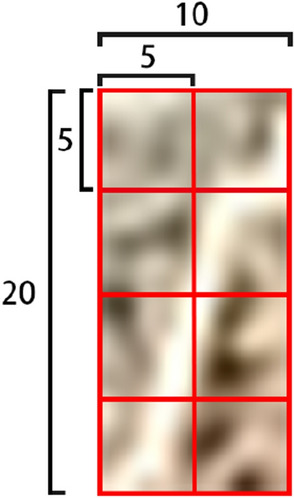
Schematic diagram of stubble image block

The local binary pattern (LBP) is the third image texture feature extraction algorithm in this article. Central pixel is located in a local area of the image, comparing the values of surrounding pixels with the central pixel. The comparison will produce a binary value, and the binary number is converted to a decimal number to replace the value of the central pixel of the image. The LBP equation is defined as follows [34]:

8 $$LBP_{P,R} = \sum \limits_{i = 0}^{P} s\left( {g_{i} - g_{c} } \right)2^{i} ,$$

9 $$s(x) = \left\{ {\begin{array}{*{20}c} {1,} & {x \ge 0} \\ {0,} & {{\text{otherwise}}} \\ \end{array} } \right.,$$

where g_c_ represents the gray value of the central pixel, g_i_ is the value of surrounding pixels. P is the total number of adjacent pixels in the local area, and R is the radius. The LBP feature of the invariant rotation mode is selected for the experiment, and the equation is defined as follows [35]:

10 $$LBP_{P,R}^{ri} = \min \left\{ {ROR\left( {LBP_{P,R} ,i} \right)} \right\},$$

where ROR (LBP_P, R_, i) represents the LBP value under different rotation directions and different starting points. The principle is shown in Fig. 7. No matter how many degrees the image is rotated, the smallest binary number is uniquely constant. The radius of the selected circular neighborhood is R = 1, and the total number of adjacent pixels P = 8. For a sample image of 10 * 20 pixels, the image is divided into eight sub-regions. As shown in Fig. 8, the size of each sub-region is 5 * 5, combined with the LBP histogram of each sub-region, the LBP texture feature vector is used as a feature of image classification.

### Classification model and evaluation index

Using three classification models of Random Forest (RF) [36], Support Vector Machine (SVM) [37], BP Neural Network (BPNN) [38] to classify stubble, residual film and broken leaves between the rows. Sample sets are divided into a training set and test set according to 8:2.

The effect of image detection is represented by accuracy (symbol: Ac), sensitivity (symbol: Se) and specificity (symbol: Sp) [39, 40]. Accuracy refers to the ratio of correctly classified samples to all samples. Sensitivity refers to the correct proportion of positive sample classification. Specificity refers to the correct proportion of negative sample classification. The calculations method for each index is shown in Table 3. All indicators calculated in the following text are obtained by tenfold cross-validation. True positive (symbol: TP) indicates the number of positive samples that are correctly classified, false positive (symbol: FP) indicates the number of negative samples that are incorrectly classified as positive samples. True negative (symbol: TN) indicates the number of negative samples that are correctly classified, false negative (symbol: FN) indicates the number of positive samples that are incorrectly classified as negative samples. In the article, the stubble is a positive sample, the broken leaves between rows and residual film are negative samples. High sensitivity means excellent stubble detection effect. High specificity means that the noise classification effect is brilliant, and more noise can be removed. In the test phase, all metrics should have high values.

**Table 3:** Calculation equation of evaluation index

Index	Equation
Accuracy	$Ac = \frac{TP + TN}{{TP + TN + FP + FN}}$
Sensitivity	$Se = \frac{TP}{{TP + FN}}$
Specificity	$Sp = \frac{TN}{{TN + FP}}$

## Results and discussion

### Classification mode and classifier performance

Table 4 shows the different results of classification using three classifiers: RF, BPNN, and SVM. The classification features are related to texture features extracted by GLCM, GLRLM, and LBP algorithms. From the overall accuracy of the model. Using GLCM combined with BPNN classifier reached 89.4%, the overall accuracy is the best among all classification categories. In terms of the stubble detection accuracy (sensitivity), the sensitivity of GLCM combined with the BPNN classifier is still the best performers in all categories. From the processing time of the algorithm, the GLCM feature is far better than other features. Although the specificity of the GLCM combined with RF is the best among all categories and its accuracy and processing time are not much different from the GLCM combined with the BPNN, we take the combination of GLCM and BPNN as the best combination of the stubble classification model. The focus of this article is the stubble detection rate. Under GLCM feature classification, the sensitivity of BPNN classification model is better than RF.

**Table 4:** Classification results of different classifiers and different texture features

Features	Model		Accuracy (%)	Sensitivity (%)	Specificity (%)	Time (per/ms)
GLCM	RF		89	87.5	**97.3**	43.5
	BPNN		**89.4**	**93.8**	96.6	**43.4**
	SVM	Linear	88.1	90.6	95.9	44.1
		Polynomial	87.1	90.6	95.2	44.5
		RadialBasis	88.1	89.1	95.9	45.1
		Sigmoid	81.4	82.8	95.9	45.1
GLRLM	RF		60.5	32.8	89	1080.1
	BPNN		67.6	37.5	93.2	1080.6
	SVM	Linear	64.3	35.9	89.7	1084.2
		Polynomial	54.3	28.1	84.2	1085
		RadialBasis	60.5	28.1	86.3	1086.5
		Sigmoid	48.6	18.8	87	1086.3
LBP	RF		46.1	23.4	87	1391
	BPNN		46.7	24.6	79.9	1386
	SVM	Linear	44.8	18	82	1397
		Polynomial	45.2	17.3	85.1	1397.7
		RadialBasis	46.9	14.8	89.5	1398.8
		Sigmoid	44.2	21.7	78.1	1398.8

Significance of the bold value indicate the maximum value of the index

In this Table 4, it is worth noting that the classification effects based on GLRLM and LBP features are very weak. In previous studies, when using GLRLM texture features for classification tasks, there is good performance [41, 42]. Khojastehnazhand et al. [43] used GLRLM and GLCM features to classify raisins and found that the classification effect of GLRLM was better than GLCM features. In this article, the reason for the poor performance of GLRLM maybe because there are too many redundant features between the features, it will adversely affect the classification and result in low classification accuracy. Through experiments, Xu et al. [44] found that the classification accuracy of GLRLM is not ideal when using full features for classification. After feature selection removes redundant features, the classification accuracy of GLRLM can be greatly improved under the same classifier. The main reason for the low effect of classification based on LBP features is the selection of blocks. After the blocks are divided, there maybe more similarities between the features of each sub-block. For example, after the 10 * 20 sample image of the stubble is divided into blocks, its sub-block images may include soil and residual film. The high feature similarity between the sub-blocks leads to a decrease in the overall classification accuracy. Comparing several blocks mode combined with sample images of different scales can improve the accuracy of LBP features in stubble classification.

The processing time of a single image with GLRLM and LBP features is another noteworthy factor. In this article, the purpose of stubble detection is to fit and extract the navigation line. There are certain requirements for real-time navigation during field operations. The time under GLCM feature classification are far superior to GLRLM and LBP features, which is also a critical factor in selecting GLCM features in this article. Therefore, for the improvement of classification accuracy of GLRLM and LBP features in stubble detection, this article does not do follow-up research.

**Fig. 9:**
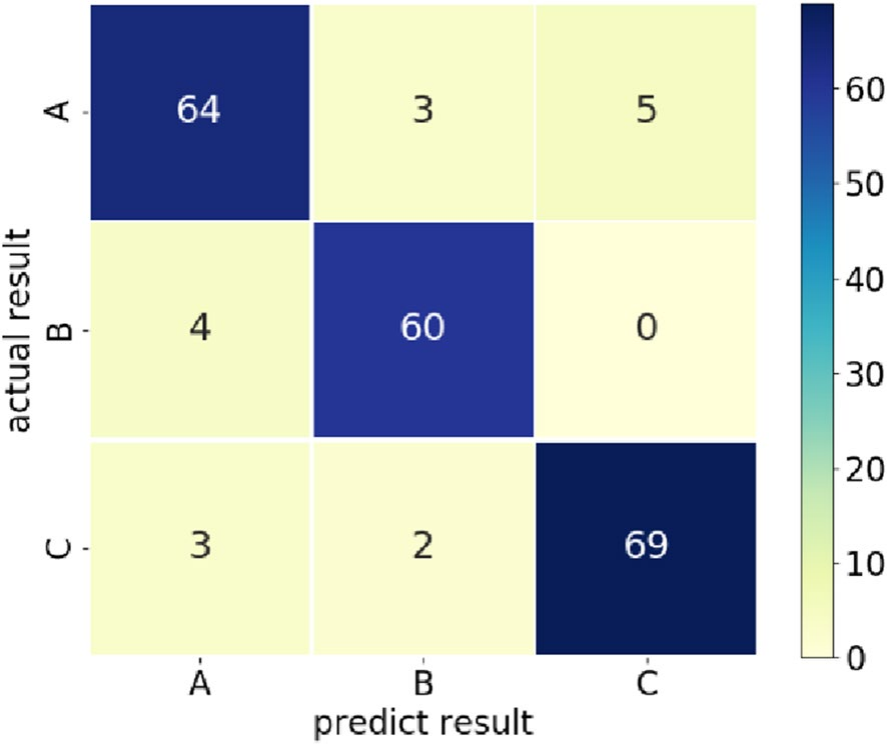
classification confusion matrix based on GLCM characteristics and BPNN classification model

Figure 9 shows the confusion matrix of the best results obtained for further study (A is the soil and broken leaves between rows, B is the stubble, C is the residual film). It can be seen from the confusion matrix in Fig. 9, the classification result of the positive sample B is acceptable. Four of the 64 images are misclassified as A. The classification effect of negative sample A is weaker than B and C because the image of the soil and broken leaves between rows is the noisiest. Its 10 * 20 pixel box contains noise that does not belong to this type of image, such as some residual film or broken straws with similar texture characteristics to stubble. Having features for other kinds of images in the sample images is also the reason for the incorrect classification of B and C images. In addition, the small training sample size is another major cause of classification errors. Although the images we collected include as many factors as possible weather conditions, camera angles, sun exposure angles, etc., while some of the image data under “special circumstances” are the key factors affecting the increase in accuracy, such as shadows, abnormal driving (the stubble changes from “upright” to “oblique”), and images under camera shake (the captured image information becomes blurred), etc. Although these scenes seldom appear in actual operations, adding more “abnormal” scene images for training in the training set can effectively enhance the accuracy and generalization of the model.

### GLCM feature performance measurement based on wavelet transform

To decompose the sample image by two-dimensional discrete wavelet transform can obtain the approximate coefficient and the detail coefficient in the three directions of horizontal, vertical and diagonal. Different texture information can be extracted from the decomposed coefficients to classify sample images. However, the wavelet family has different mother wavelets, and different mother wavelets have different effects on image decomposition. There is currently no documented record of the best basis wavelet for stubble detection. To determine the ideal wavelet suitable for the three types of image classification problems, several mother wavelets can be tested through a trial and error process [45]. For this reason, 22 widely used wavelets are selected as the basis wavelet set to choose the best basis wavelet [46, 47], which includes Daubechie (db) series (db1, db2, db3, db4, db5, db6, db7, db8, db9, db10), Symlet series (sym2, sym3, sym4, sym5, sym6, sym7, sym8), and Coiflet series (coif1, coif2, coif3, coif4, coif5). To decompose the image through different basis wavelets, record the approximate coefficients and the detail coefficients, comparing the classification effect by extracting the GLCM features of the image. The classifier uses the BPNN, because it performs best in experiments of GLCM features for classification.

**Fig. 10:**
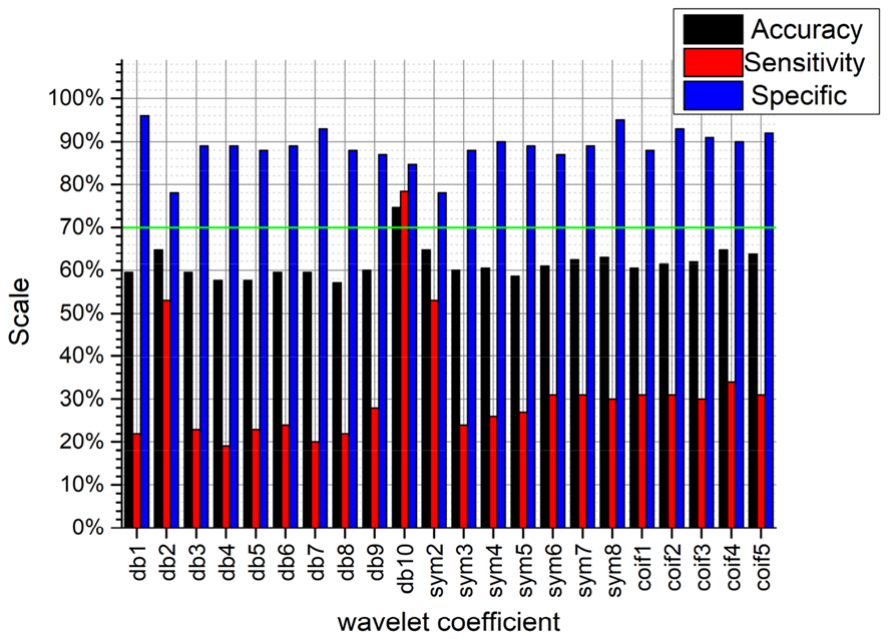
Classification performance of different base wavelets under approximate images

**Fig. 11:**
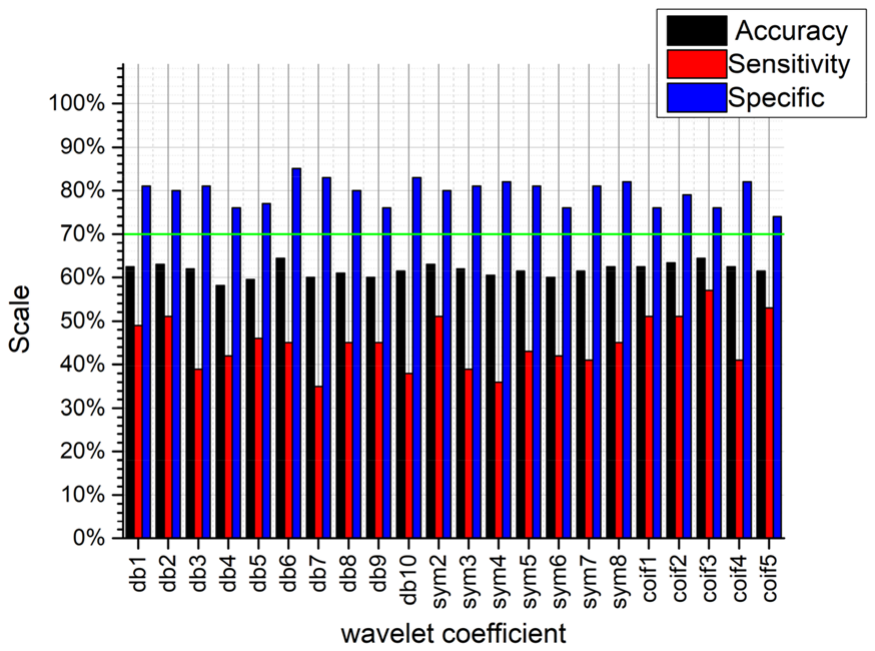
Classification performance of different base wavelets under horizontal detail images

**Fig. 12:**
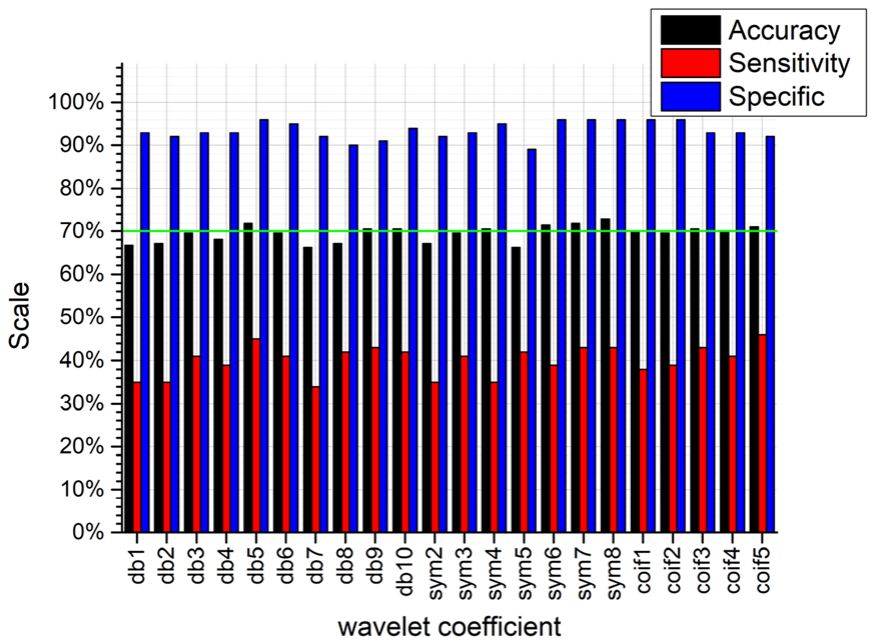
Classification performance of different basis wavelets under vertical detail images

**Fig. 13:**
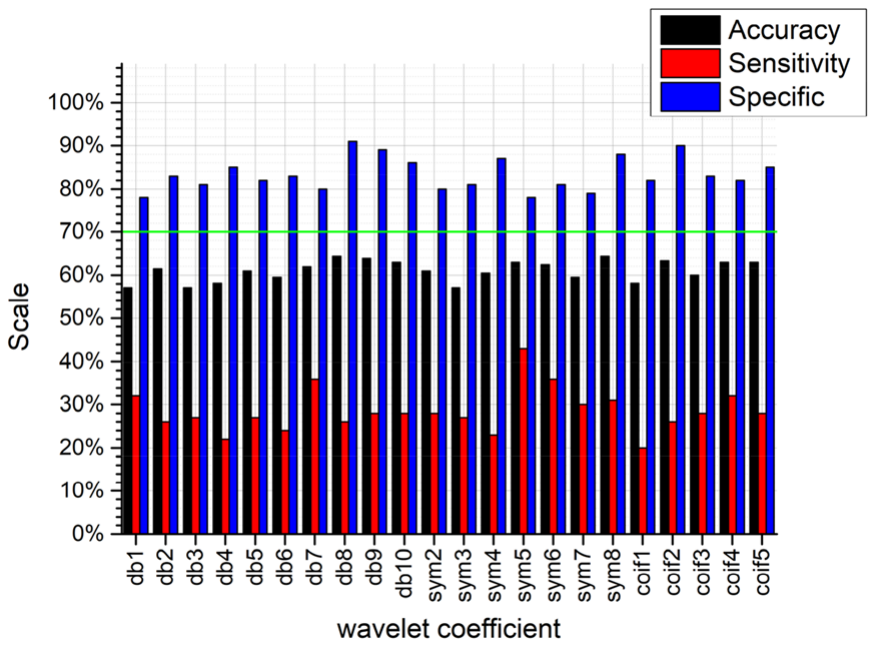
Classification performance of different base wavelets under diagonal detail images

It can be seen from Figs. 10, 11, 12, and 13, no matter which wavelet base is selected, the sub-band coefficients in the approximate, horizontal, and diagonal directions are lower than the classification effect under the vertical coefficient. After wavelet transform, the vertical coefficients retain the part of the image that changes drastically in the vertical direction. Although there is much noise interference in the field images of the residual film recovery, the vertical characteristics of the stubble remain in the whole image of the stubble sample. The accuracy of the vertical decomposition coefficients is better than other three wavelet coefficients. The classification accuracy of multiple wavelet-based decomposition coefficients is above 70%.

In general, the classification accuracy of wavelet coefficients after wavelet decomposition is lower than original image. However, the texture features extracted from the wavelet decomposition coefficients can be used as extended features. The classification accuracy can be improved by fusing with the texture features of the original image. We use 70% as the classification accuracy threshold (the green lines in Figs. 10, 11, 12, and 13 are the accuracy selection threshold), selecting features with classification accuracy greater than 70% to combine with the original image texture features to verify the fusion features classification effect. The approximate coefficients decomposed by db10 and the vertical coefficients decomposed by db5, db9, db10, sym4, sym6, sym7, sym8, coif1, and coif3 are selected. The classification effect of the selected coefficients is shown in Table 5.

**Table 5:** Classification accuracy under different wavelet basis decomposition coefficients

Wavelet basis	Accuracy/%
db10 (approximate coefficient)	74.6
db5 (vertical factor)	71.9
db9 (vertical factor)	70.5
db10 (vertical factor)	70.5
sym4 (vertical factor)	70.5
sym6 (vertical factor)	71.4
sym7 (vertical factor)	71.9
sym8 (vertical factor)	72.9
coif1 (vertical factor)	70
coif3 (vertical factor)	70.5

Combining the GLCM texture features extracted from the wavelet coefficients and original image as a new feature. There are totals of 48-dimensional features. The classifier chooses the best BPNN in the original image classification. The Table 6 shows the classification effect of fusion features on images. From the perspective of accuracy, the performance of the fusion feature is better than the texture feature of the single original image. Among the different wavelet bases, the coif3 wavelet is the wavelet base with the highest accuracy. Its fusion feature accuracy rate is 93.2%. Compared with the original image classification accuracy rate of 89.4%, its accuracy rate is increased by 3.8%. Sensitivity and specificity indicators are worth noting. The performance of some fusion features is lower than the single feature of the original image. However, the performance of the three indicators of the coif3 fusion feature is better than single texture feature, which also verifies the hypothesis that the texture feature of the wavelet decomposition image and original image can improve the classification effect.

**Table 6:** Classification effect of fusion features

Selected image	Accuracy (%)	Sensitivity (%)	Specificity (%)
Original image + db10 (approximate coefficient)	91.7	91.9	96.3
Original image + db5 (vertical factor)	92.3	94.6	98.5
Original image + db9 (vertical factor)	92.5	93.2	97.1
Original image + db10 (vertical factor)	92.1	97.3	97.1
Original image + sym4 (vertical factor)	90.5	94.6	96.3
Original image + sym6 (vertical factor)	91.4	97.3	97.1
Original image + sym7 (vertical factor)	91.9	97.3	**98.5**
Original image + sym8 (vertical factor)	91.8	95.9	97.1
Original image + coif1 (vertical factor)	91.9	95.9	97.8
Original image + coif3 (vertical factor) (Additional file 1)	**93.2**	**98.6**	97.8

Significance of the bold value indicate the maximum value of the index

### Evaluation of classification effect of fusion features

**Fig. 14:**
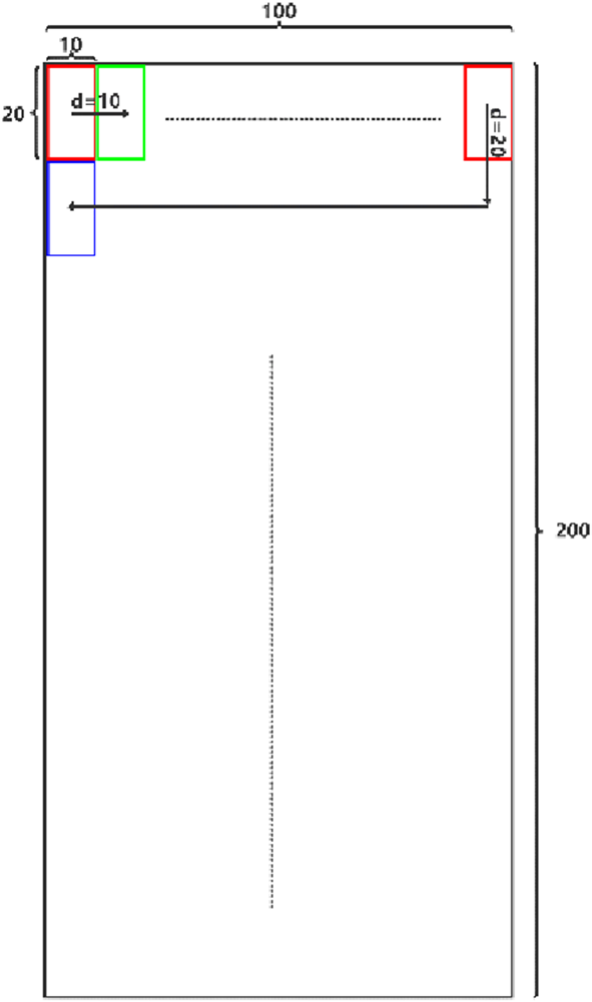
The principle of row by row and block by block scanning

It selects three types of field images from different locations, different periods, and abnormal driving to test the algorithm. The size of the image collected by the camera is 640 × 480. We extract the stubble row facing the tractor driver as the detection area (100 × 200 ROI), and scan the ROI area row by row and block by block. The process is shown in Fig. 14. The images judged by the classifier as stubble are framed in red on the image, and the detection results will be given in the following sections.

#### (1) Image detection results of different locations

**Fig. 15:**
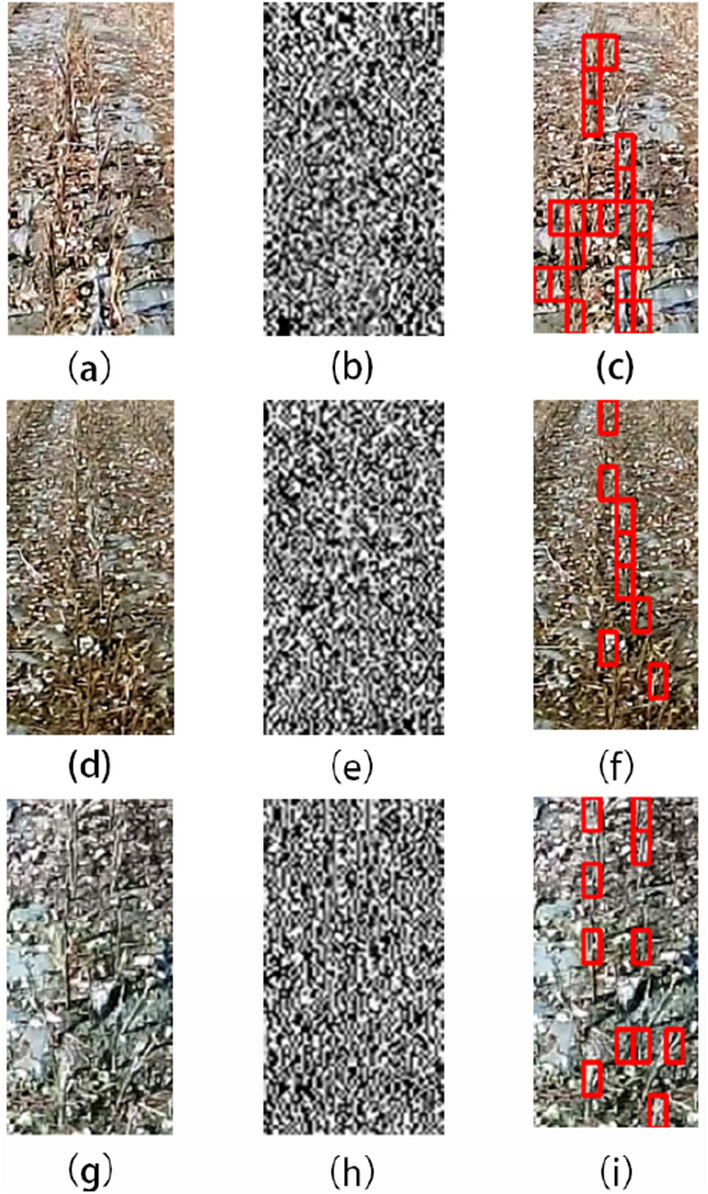
Detection results at different locations: **a**, **d**, **g** are the original images; **b**, **e**, **h** are the vertical coefficients after coif3 wavelet decomposition; **c**, **f**, **i** are algorithmic detection result

We selected field images from different locations for algorithm detection. The main differences are the color of the soil, the distribution of broken leaves and broken stalks. All images can detect the stubble, and some images have leak detections and error detections. Figure 15a, d, g is selected as representative images of the three types of places. From the detection results, the detection results (a) are acceptable because of its obvious stubble characteristics and low noise interference. For the image (d), the cotton shells and broken leaves are distributed in large quantities. These two types of noise are similar in color to the stubble, it is easy to cover up and disturb the texture information of the stubble. For the image (g), the detection effect of oblique stubble and stubble in shadow is weak. For different locations, the overall detection rate of stubble is acceptable. The color of the soil and broken leaves will not affect stubble detection, but when the noise of broken leaves is considerable, it will miss stubble detection. In addition, for irregularly shaped stubbles, the algorithm is likely to missing detection, which is the main reason for stubble detection errors in different locations.

#### (2) Image detection results in different periods

**Fig. 16:**
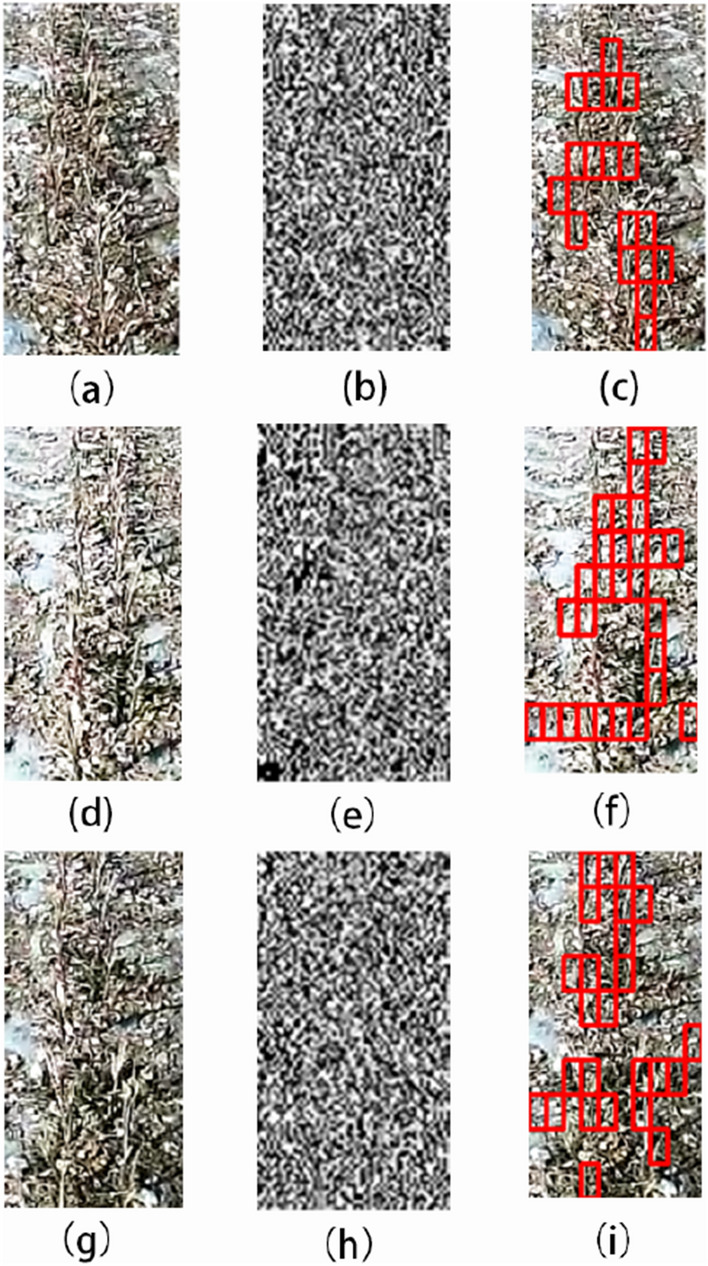
Detection results at different time periods: **a**, **d**, **g** are the original images collected in the morning, noon, and evening at the same location; **b**, **e**, **h** are the vertical coefficients after coif3 wavelet decomposition; **c**, **f**, **i** are the algorithm detection results

We selected field images in different periods for algorithm detection. The images were from the same place, and the periods were mainly morning, noon and afternoon. Figure 16a is an image of the place in the morning. The influence of morning light is not obvious. The stubble in the image can basically be detected by the algorithm, and the effect is good. Figure 16d is an image of the place at noon. The light makes the whole image brighter, and the characteristic information of the stubble is not obvious due to the sunlight, but the overall detection effect is within an acceptable range. The influence of the shadow caused by the light is worth noting. The stubble in the shadow and the shadow of the stubble are easy to cause error detection. Figure 16g is an image of the place in the afternoon. The light is not as intense as noon, and the overall detection effect of stubble is better than the detection result at noon.

#### (3) Detection results of abnormal driving images

**Fig. 17:**
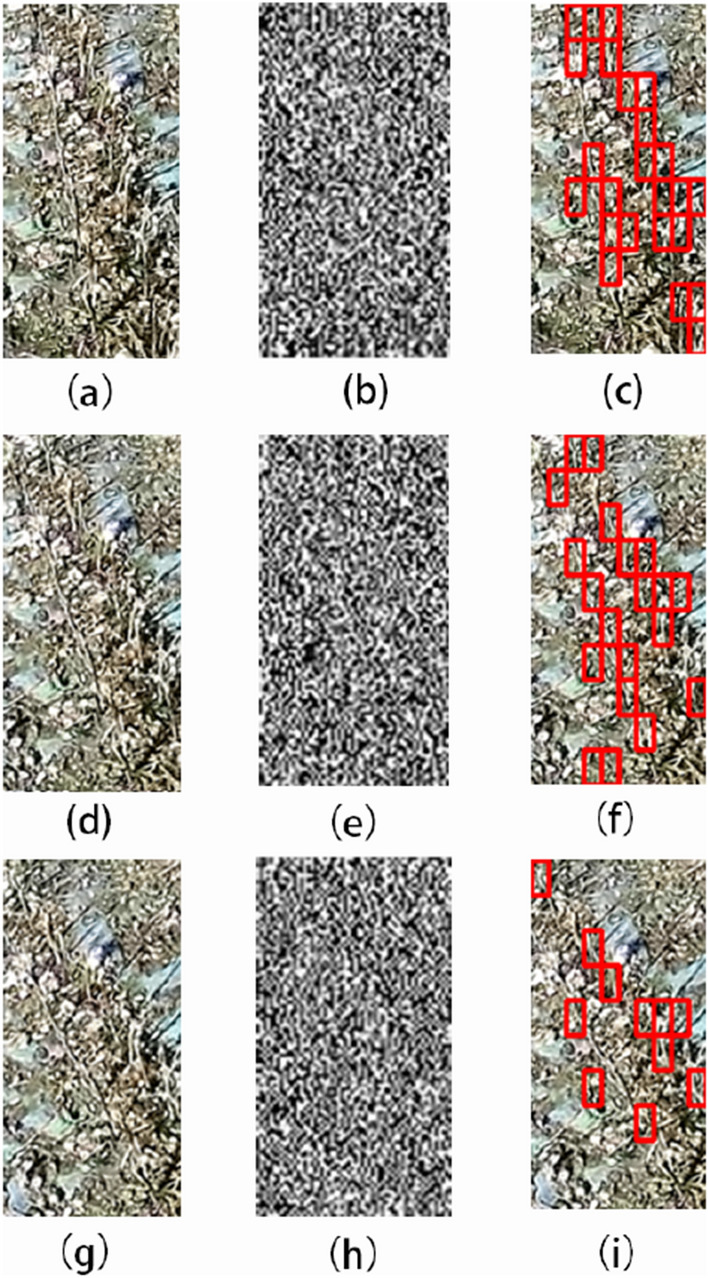
Abnormal driving detection results: **a**, **d**, **g** are the driving states of the tractor with the small deviation from the course, the medium deviation from the course and the large deviation from the course, respectively; **b**, **e**, **h** are the vertical coefficients after coif3 wavelet decomposition; **c**, **f**, **i** are the algorithm detection results

We selected abnormal driving images for algorithm detection. Abnormal driving refers to the driver’s mistaken driving. The tractor is collecting images while deviating from the residual film recovery route. Figure 17a, d are images with small and medium deviations, respectively. In these two cases, the stubble image can be successfully detected. Figure 17g is an image under a large deviation. The tractor deviates too much from the normal route, the collected stubble changes from a vertical state to a sloping state, and the inclination angle is large. In this case, the stubble detection is prone to missing detection. The main reason is the lack of inclined stubble samples in the sample data set for model training.

#### (4) Different algorithm detection results

**Fig. 18:**
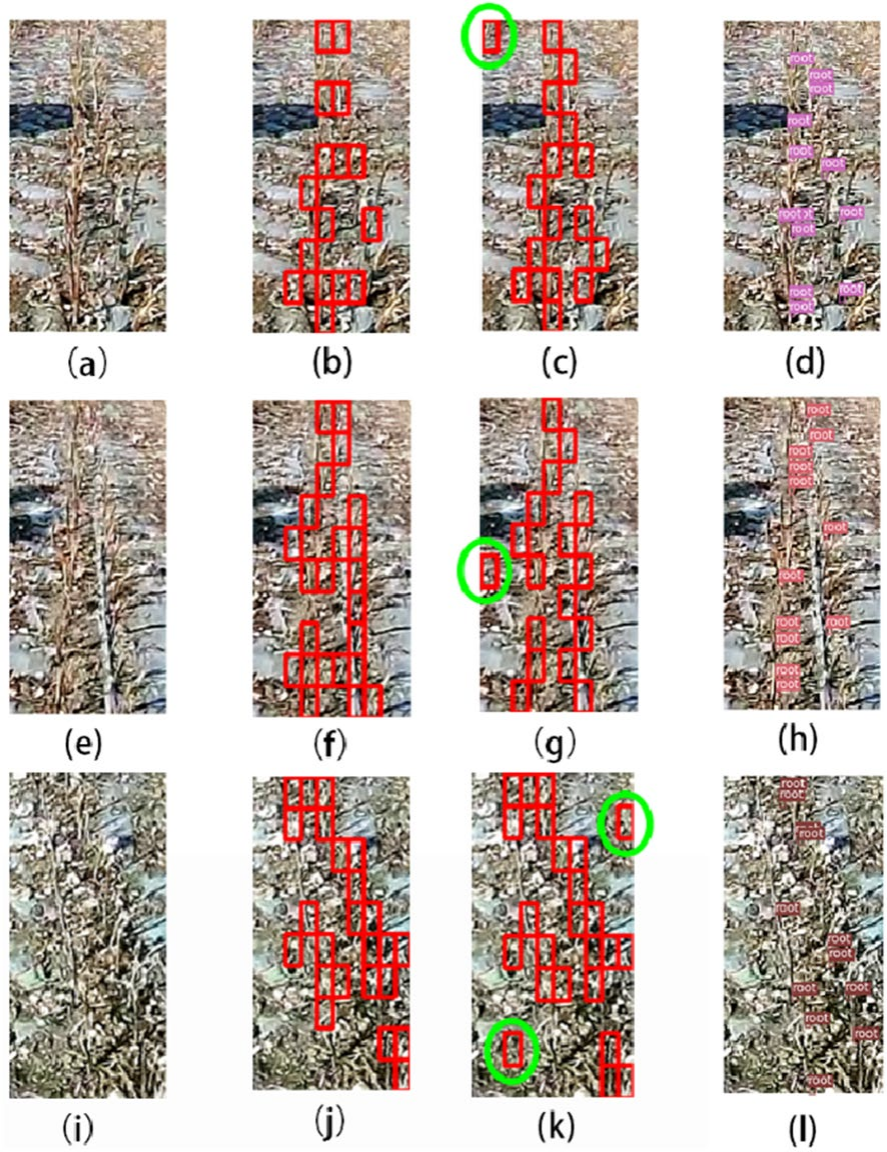
Algorithm comparison results: **a**, **e**, **i** are original images; **b**, **f**, **j** are fusion feature detection results; **c**, **g**, **k** are GLCM feature detection results; **d**, **h**, **l** are YOLOv3 detection results

To further verify the effect of the fusion feature in stubble detection, the GLCM feature and YOLOv3 algorithm were selected to carry out a comparative experiment on stubble detection. In the comparative experiment, the fusion feature and GLCM feature both choose the best BPNN as the classification model. YOLOv3 uses the original Draknet-53 network, by marking 2000 stubble images as a training set, training 4000 epochs after the model Loss value stable at 0.1 near, and finally selected the training 6000 epochs of the model as the best detection model of YOLOv3. The comparison results are shown in Fig. 18a, e, i is the original image. For fusion features, the stubble is basically detectable, and there is no obvious error detection, the effect is as (b), (f), (j) shown. For the GLCM feature, there is a partial missing detection in the stubble detection, but compared with the fusion feature, there is clear error detection, as shown in figure (c), (g), (k). The green frame indicates that other types of images are error detected as stubble, and the error detected images would have a severe impact on the later navigation line extraction.

The detection result of YOLOv3 has no error detection, but the stubble missing detection is serious. The small training sample is main reason, and the second is the size of the stubble. Most of the stubble belongs to small targets. In the deep network detection framework easy to miss the small target, need further improvement of the network to improve the detection effect. In general, the stubble detection based on fusion features is better than the comparison algorithm. It can complete the task of stubble detection in a complex background, thus providing the basis for the next step of residual film recovery navigation line extraction.

## Conclusion

The stubble detection in the natural environment is essential for the navigation line fitting of residual film recovery operations. The following conclusions can be drawn from the test results:

1. It is necessary to manually select stubble, residual film, soil and broken leaves to make a training set and testing set. Compared with the previous image processing technology, this method is more targeted and complexity of the overall image will not affect the extraction of stubble features.
2. For the classification of soil and broken leaves, stubble, residual film. The texture feature based on the GLCM has considerable reference value. From the time point of view, GLRLM and LBP are not suitable for the requirements of detecting stubble in navigation operations.
3. Compared with the three typical classifiers of RF, SVM and BPNN, it can be found that the classification effect of BPNN combined with GLCM texture features is the best of all types.
4. The classification effect of texture features under wavelet coefficients is discussed. It is found that the classification effect of the wavelet coefficient texture feature is not as good as the classification feature of the original image, but the texture feature extracted from the wavelet coefficient can be used as a supplementary feature, combining with the texture feature of the original image can improve the classification effect. By comparing the commonly used wavelet bases, it can be found that the coif3 wavelet coefficient texture combined with the original image texture will have the best performance. Compared with the original image texture feature, its accuracy rate increases by 3.8%, the sensitivity increases by 4.8%, the specificity increased by 1.2%, and a better detection effect was achieved.

The results show that the texture feature combined with wavelet decomposition is an effective stubble detection feature. Compared with the previous single image features, the fusion feature detection in this article has a higher accuracy rate. It provides a model reference for the detection of stubble of different crops, and a reliable technical support for the subsequent visual navigation of residual film recovery. It is worth noting that the speed of stubble detection using this type of fusion feature is slow. Our future work will focus on optimizing the detection algorithm to reduce the detection time.

## Supplementary Information

**Additional file 1.** Original image + coif3 (vertical factor).

## Data Availability

All data and material generated or analysed during this study are included in this published article.
